# Role of FruR transcriptional regulator in virulence of *Listeria monocytogenes* and identification of its regulon

**DOI:** 10.1371/journal.pone.0274005

**Published:** 2022-09-02

**Authors:** Hossam Abdelhamed, Reshma Ramachandran, Lakshmi Narayanan, Shamima Islam, Ozdemir Ozan, Nancy Freitag, Mark L. Lawrence

**Affiliations:** 1 Department of Comparative Biomedical Sciences, College of Veterinary Medicine, Mississippi State University, Starkville, MS, United States of America; 2 Department of Poultry Science, Mississippi State University, Starkville, MS, United States of America; 3 Department of Pharmaceutical Sciences, University of Illinois at Chicago, Chicago, Illinois, United States of America; LSU Health Sciences Center School of Dentistry, UNITED STATES

## Abstract

*Listeria monocytogenes* is a ubiquitous opportunistic foodborne pathogen capable of survival in various adverse environmental conditions. Pathogenesis of *L*. *monocytogenes* is tightly controlled by a complex regulatory network of transcriptional regulators that are necessary for survival and adaptations to harsh environmental conditions both inside and outside host cells. Among these regulatory pathways are members of the DeoR-family transcriptional regulators that are known to play a regulatory role in sugar metabolism. In this study, we deciphered the role of FruR, a DeoR family protein, which is a fructose operon transcriptional repressor protein, in *L*. *monocytogenes* pathogenesis and growth. Following intravenous (IV) inoculation in mice, a mutant strain with deletion of *fruR* exhibited a significant reduction in bacterial burden in liver and spleen tissues compared to the parent strain. Further, the Δ*fruR* strain had a defect in cell-to-cell spread in L2 fibroblast monolayers. Constitutive activation of PrfA, a pleiotropic activator of *L*. *monocytogenes* virulence factors, did not restore virulence to the Δ*fruR* strain, suggesting that the attenuation was not a result of impaired PrfA activation. Transcriptome analysis revealed that FruR functions as a positive regulator for genes encoding enzymes involved in the pentose phosphate pathway (PPP) and as a repressor for genes encoding enzymes in the glycolysis pathway. These results suggested that FruR may function to facilitate NADPH regeneration, which is necessary for full protection from oxidative stress. Interestingly, deletion of *fruR* increased sensitivity of *L*. *monocytogenes* to H_2_O_2_, confirming a role for FruR in survival of *L*. *monocytogenes* during oxidative stress. Using anti-mouse neutrophil/monocyte monoclonal antibody RB6-8C5 (RB6) in an *in vivo* infection model, we found that FruR has a specific function in protecting *L*. *monocytogenes* from neutrophil/monocyte-mediated killing. Overall, this work clarifies the role of FruR in controlling *L*. *monocytogenes* carbon flow between glycolysis and PPP for NADPH homeostasis, which provides a new mechanism allowing metabolic adaptation of *L*. *monocytogenes* to oxidative stress.

## Introduction

*Listeria monocytogenes*, a facultative intracellular pathogen, is the causative agent of listeriosis, a potentially fatal foodborne infection [1]. *L*. *monocytogenes* is a frequent contaminant of food processing facilities and has been responsible for some of the largest and most expensive food recalls in US history [2–5]. Although healthy individuals generally exhibit febrile gastroenteritis, immunocompromised persons, pregnant women, and elderly persons often suffer more severe invasive forms of illness [6]. *L*. *monocytogenes* invasive disease has a high mortality rate even with antibiotic treatment [6].

*L*. *monocytogenes* has a biphasic lifestyle, transitioning from a saprophyte in the environment to a facultative intracellular pathogen within host cells. This transition is mainly mediated by virulence regulator PrfA, a member of the Crp/Fnr family [7–10]. PrfA regulates the transcription of core virulence factors necessary for survival and replication of *L*. *monocytogenes* within host environment [11,12]. PrfA activity is retained at a low basal level in the outside environment, while PrfA activity becomes high upon vacuolar escape and access to host cytosol [11,13]. Published data suggest that binding of PrfA to glutathione in the cytosol of the host cell is necessary for increasing the activity of PrfA at target genes [14].

*L*. *monocytogenes* moves through various niches and responds to each environment it encounters, including extracellular, abiotic, and intracellular environments [7,15]. For this to occur, *L*. *monocytogenes* requires a well-coordinated regulatory system that can sense and respond to a unique environment through signaling pathways that lead to the expression of proteins/factors aiding in survival [16]. These transcriptional networks are highly complex and require the coordination of a large number of regulatory elements [17,18]. To fully understand the pathogenic processes that allow *L*. *monocytogenes* to survive and adapt in stress-related environmental conditions and host colonization, it is important to clarify the function of transcriptional networks and how they are regulated. Many features and components of these transcriptional factors have not been well characterized.

Transcriptional regulators of the DeoR-family are widespread in Gram-positive and Gram-negative bacteria, and most of the characterized regulators in this family are involved in sugar metabolism or nucleoside metabolism [19,20]. However, there are some examples of DeoR-family members participating in bacterial responses to environmental conditions and stress [21–24]. Proteins of the DeoR family exhibit several common features. First, their length is highly conserved, ranging from 240 to 260 amino acids. Second, DeoR-family proteins contain a highly-conserved N-terminal region with a helix-turn-helix DNA-binding motif [22,25]. Third, the C-terminal region acts as a sensor domain and is responsible for oligomerization and binding effectors, which are generally phosphorylated intermediates of the relevant metabolic pathway [25]. By analyzing the genome of *L*. *monocytogenes* strain F2365, we found seven uncharacterized members of the DeoR-family of regulators. A phylogenetic tree of the seven DeoR-family regulators revealed that they can be classified into two main clades (S1 Fig).

One of the DeoR-family regulators encoded by *L*. *monocytogens* is FruR, which is a transcriptional repressor of the fructose operon (*fruRBA*) [26]. FruA is a fructose phosphotransferase, and FruB is phosphofructokinase [27–29]. However, FruR influences the regulation of other genes encoding enzymes involved in different pathways such as glycolysis, gluconeogenesis, and the Krebs cycle [29,30]. In *Escherichia coli* and *Salmonella typhimurium*, a *fruR* null mutation altered the rates of utilization of at least 36 carbon sources [31]. Therefore, it was concluded that FruR serves as a pleiotropic transcriptional regulator that modulates the flow of carbon through different pathways [31–34]. The function of FruR in *L*. *monocytogenes* is unexplored, even though sugar metabolism is crucial for virulence [12,35,36]. This provoked us to investigate the role of FruR in *L*. *monocytogenes* virulence and to determine the FruR regulon in *L*. *monocytogenes*.

## Material and methods

### Bacterial strains and growth conditions

*Listeria monocytogenes* F2365 strain and derivative mutants were cultured in brain heart infusion (BHI) medium (Difco) at 37°C. *Escherichia coli* DH5α was grown in Luria–Bertani (LB) (Difco), either broth or agar. Bacterial strains and plasmids used in this study are described in Table 1. Murine fibroblast (ATCC CRL-2648) cell lines were grown in Dulbecco’s Modified Eagle’s Medium (DMEM) (ATCC, Manassas, VA) supplemented with 20% fetal bovine serum (FBS) (Atlanta Biologicals, Norcross, GA) and 1% glutamine. Murine macrophage cell line J774A.1 was grown in DMEM supplemented with 10% FBS. Cultures were maintained at 37°C with 5% CO_2_ under humidified conditions. When necessary, 10 μg/ml erythromycin, 100 μg/ml ampicillin, 50 μg/ml neomycin, and/or 10 μg/ml gentamicin were added to the growth media.

**Table 1:** Bacterial strains, plasmids, and primers used in this study.

Bacterial strains, plasmid		Description	Source/Reference		
**Strains**					
*E*. *coli*					
DH5α	Competent cells				Invitrogen
*L*. *monocytogenes*					
F2365	Wildtype serotype 4b strain				[37]
NF-L1124	10403S *actA-gus-neo-plcB* (prfA* strain)				[38]
F2365Δ*fruR*	F2365Δ*fruR* mutant strain				This study
F2365Δ*fruR*::pPL2-*fruR*	F2365Δ*fruR*::pPL2-*fruR* complemented strain				This study
ΔfruRprfA*	F2365Δ*fruR*::10403S *prfA* L140F *actA-gus-neo-plcB* (prfA* strain)				This study
**Plasmids**					
pHoss1	8,995 bp, pMAD,::*secY* antisense, Δ*bgaB*, Amp^r^, Ery^r^				[39]
pPL2	6,123 bp, PSA *attPP*, Chl^r^				[40]
*pLm*Δ*fruR*	pHoss1,::Δ*fruR*, Amp^r^, Ery^r^				This study
pPL2-*fruR*	pPL2,::*fruR*, Amp^r^, Ery^r^				This study
**Primers for deletion strain and complementation (5′–3′)**					
FruR_A	AAA**GTCGAC**GGTGGTTCGCCTAGAGTCAT				*Sal*I
FruR _B	TCCATCCAAAGTCAGTTTACCA				
FruR _C	TGGTAAACTGACTTTGGATGGAATTCCTTCTGCAGTAAAAGAATCC				
FruR_D	AAA**CCATGG**ATGCAGTAGCTCCACCTGTTG				*Nco*I
FruR_seq	GCATGTGTAAGCATACTGTGT				
FruR _Comp_F01	AAA**GAGCTC**TGTTTTTGAATGAAAACGGTTG				*SacI*
FruR_Comp_R01	AAA**GTCGAC**GACCGCCAAGATTTAATTGG				*Sal*I
**ActA promoter primers**					
5’actA	GATGCTTCTAAAAAAGTTGCTGAAGC				[38]
3’actA	TATTCATGAATTATTTTAAGAATATCA				[38]
**Primers for qPCR (5′–3′)**					
FruA_F	ATCACAGGTGCGGTAGTAATG				
FruA_R	CAAGGTTGGGATACAAGGAAGA				
FruB_F	GTGCCTCCTTCACTTGGAAAT				
FruB_R	AGCAGTTCTTGACCAGTTGTATC				
Glck_F	CAGCTGGTGAAATTGGGCAT				
Glck_F	TCGCTACTCGAACGATTCCT				
RsbT_F	GGGACGATTGACACGGAAAG				
RsbT_R	ACAAGTCTAACCGCTTCGGA				
16S_F	CAAGCGTTGTCCGGATTTATTG				
16S_R	GCACTCCAGTCTTCCAGTTT				

In the above primer sequences, bold face indicates restriction enzyme sites, and underlined text indicates overlap to FruR _B primer. Amp^r^, ampicillin resistant; Ery^r^, erythromycin resistant; Chl^r^, chloramphenicol resistant.

### Construction of Δ*fruR* mutant and complemented strain

The *fruR* gene (LMOf2365_2307) encoding fructose operon transcriptional repressor was targeted for in-frame deletion using splice overlap extension and allelic exchange with pHoss1 as previously published [39]. The sequence of primers used for construction and validation of the mutant is listed in Table 1. PCR analysis with flanking primers (A and D) was done to confirm deletion of the *fruR* gene. The *fruR* deletion was further confirmed by sanger sequencing of the amplified deletion fragment. For complementation assays, a DNA fragment containing the entire *fruR* gene and its upstream promoter was amplified from the F2365 genome and ligated into pPL2 shuttle integration vector [40]. The *fruR*-complementing plasmid was electrotransformed into F2365Δ*fruR* to obtain the complemented strain. Successful complementation was confirmed by PCR analysis and sequencing of genomic DNA.

### Growth assay

Growth of wildtype F2365, F2365Δ*fruR*, and the complemented strain in BHI broth and minimal medium (MM) were compared [41]. Bacterial growth curves were generated by inoculating media with a standard dose of overnight culture in a 24-well plate and incubated on a Cytation 5 Cell Imaging Multi-Mode Reader (BioTek) at 37°C under static growth conditions. The optical density at 600 nm (OD_600_) was monitored automatically every hour for 24 hours with moderate shaking (160 rpm) for 15 seconds before reading. The growth assays were conducted in three independent experiments, and each experiment was run with four replicates. Growth curves with different carbon sources were obtained by inoculating the strains into MM supplemented with 50 mM glucose, mannose, fructose, maltose, or sucrose.

### Virulence in mice

All animal experiments were done in accordance with a protocol (18–508) approved by the Institutional Animal Care and Use Committee at Mississippi State University. The virulence of Δ*fruR* was compared with *L*. *monocytogenes* strain F2365 and complemented strain in BALB/c mice (Jackson Laboratory) through intravenous (IV) injection via the tail vein and through oral routes as previously described [42–44]. Twelve-week-old female BALB/c mice were housed in 5 cages (5 mice/cage) and kept under specific pathogen-free conditions. After prewarming with a heat pad, each mouse was infected with approximately 2 x 10^4^ CFU/mouse in 200 μl volume for each bacterial strain. Control mice were inoculated with sterile saline. Animals were monitored daily for signs of disease. At 24- and 72-hours post-infection, mice were euthanized. After euthanasia, spleen and liver tissues were aseptically removed, weighed, and homogenized in sterile saline. Serial dilutions of homogenized tissues were spread on BHI agar plates to determine bacterial concentration in the tissues. For oral infection studies, approximately 5.5 × 10^6^ CFU/ml of each bacterial strain was orally infused by gavage needle in each mouse and mice were euthanized on day 5 post-infection to determine the number of CFU per gram of tissue.

### PrfA* expression in Δ*fruR*

The constitutively activated form of PrfA (PrfA*) was introduced into Δ*fruR* strain using U153 bateriophage-mediated phage transduction as described [45,46]. Briefly, U153 phage were grown in the presence of *L*. *monocytogenes* strain NF-L1775 containing the transcriptional fusion fragment (prfA* actA-neo-gus); the resulting phage lysate was incubated with the Δ*fruR* strain for 40 min at room temperature. After incubation, the mixture was spread on BHI plates containing 50 μg/ml neomycin and 50 μg/ml 5-bromo-4-chloro-3-indolyl-β-d-glucuronide (Inalco, Milano Italy). Positive transductants that were both neomycin resistant and dark-blue in color (conferred a high level of prfA* actA-neo-gus expression and activity) were isolated and used in further analysis. The integration of prfA* actA-neo-gus fragment in the chromosome of the Δ*fruR* strain was then reconfirmed by PCR analysis using *actA* promoter primer (Table 1) and sequencing of amplified product. Virulence of the resulting ΔfruRprfA* strain was evaluated in BALB/c mice using IV injection with 200 μl of 10^4^ CFU/ml. Spleens and livers were harvested after 72 h, and bacterial CFUs were determined.

### Plaque assay in murine L2 fibroblasts

Plaque assays using murine fibroblast (ATCC CRL-2648) monolayers were performed as described [47]. Briefly, fibroblasts were grown in DMEM in six-well tissue culture plates until they reached confluence. Confluent monolayers were infected with wildtype F2365, F2365Δ*fruR*, and the complemented strain for 1 hour with a multiplicity of infection (MOI) of approximately 1 to 10. DMEM agar containing 10 μg/ml of gentamicin was added to each well, and plates were incubated at 37°C and 5% CO_2_. After 96 hours, plaques were visualized by adding an overlay consisting of DMEM, 0.5% agarose, and 0.2% neutral red. The plates were stained overnight, and plaque size was evaluated using ImageJ from at least three independent experiments.

### Infection of macrophages

Macrophage cell line J774A.1 was infected with wildtype F2365, F2365Δ*fruR*, and the complemented mutant strain at a mean MOI of 1 to 10. After infection, extracellular bacteria were killed by adding 0.5 ml DMEM medium containing 10 μg/ml gentamicin. After incubation for 2, 4, and 6 hours, infected cells were washed and lysed, and lysate was spread on BHI agar plates to quantify intracellular bacteria.

### RNA-seq analysis

*L*. *monocytogenes* wildtype F2365 and Δ*fruR* (four independent biological replicates) were grown in BHI at 37°C until the growth reached mid-logarithmic phase (OD_600_ reached ∼ 0.06). Bacterial pellets were collected by centrifugation. The resulting pellet was maintained on ice and placed into RNase-free tubes (ThermoFisher Scientific) that contained 10 volumes of RNA*later* solution (Ambion, Austin, TX) to minimize RNA degradation. RNA was isolated using a FastRNA™ SPIN Kit for Microbes with a FastPrep-24™ (MP Biomedicals, Santa Ana, CA) following the manufacturer’s instructions. Genomic DNA was eliminated from total RNA using on-column DNase treatment with an RNase-Free DNase Set (QIAGEN, Hilden, Germany). The quantity and quality of total RNA were analyzed using a NanoDrop ND-1000 spectrophotometer (Thermo Scientific, USA) by measuring the OD260 nm/OD280 nm ratio. RNA samples (obtained in triplicate) were submitted to Novogene Corporation for quality control, library preparation, and sequencing using the HiSeq platform (Illumina) as described [48]. Before analyzing data, raw reads were filtered to remove reads containing adapters or low-quality reads. Filtered reads were mapped to the F2365 genome using Bowtie2 [49]. Transcriptomic analysis was conducted using Bioconductor Edge R [50] to identify differentially expressed genes of the wild type compared to mutant strain. To minimize false-positive results, a stringent False Discovery Rate (FDR) cutoff of 1 was applied.

### Verification of gene expression by quantitative real-time PCR (qRT-PCR)

To validate RNA-seq results, qRT-PCR was performed on the same RNAs used for RNA-seq. Primers and gene information are listed in Table 1. cDNA was synthesized from RNA (5 μg) using the SuperScript cDNA synthesis kit (Thermo Scientific). The product of first-strand cDNA synthesis was diluted 50 times, and qRT-PCR was performed in a 20 μl reaction containing SYBR Green Real-time PCR master mix (Roche Diagnostic GmbH, Mannheim, Germany). Amplification and detection of specific products were performed with the Mx3000P™ real-time PCR system (Stratagene) with the following cycle profile: initial denaturation at 95°C for 10 min, 40 cycles of 95°C for 30 s, 55°C for 30 s, and 72°C for 1 min. Expression of each gene was normalized against the expression of 16S rRNA before comparative analysis. For each gene, triplicate assays were performed, and transcription levels were quantified by 2^-△△CT^ method [51].

### Hydrogen peroxide sensitivity

Growth and survival of the Δ*fruR* strain were compared to wildtype during growth in BHI medium at 37°C supplemented with different concentrations of H_2_O_2_. Growth and survival experiments were performed on exponentially growing bacteria (OD_600_ < 0.2), which were harvested by centrifugation and resuspended in BHI supplemented with different concentrations of H_2_O_2_. Hydrogen peroxide was added to BHI to a final concentration of 10, 20, 50, and 100 mM. For the growth experiments, OD_600_ values were automatically determined every hour for 24 hours at 37°C using a Cytation 5 Cell Imaging. In the survival experiments, CFUs were quantified for wildtype, Δ*fruR*, and the complemented mutant strain at zero time and after 1 hour incubation with different concentrations of H_2_O_2_. The survival rate of bacteria was determined by comparing CFUs after treatment with H_2_O_2_ with the initial number of CFUs for the same strain present before treatment (time point 0).

### Δ*fruR* virulence in neutrophil/monocyte depleted mice

Female Balb/c mice aged 10–12 weeks were neutrophil/monocyte depleted using intraperitoneal injection with monoclonal antibody RB6-8C5 (RB6) (500 μg) at 24 and 6 hours before initiation of bacterial infection. Neutrophil/monocyte depletion was microscopically confirmed using peripheral blood of the mice. Neutrophil/monocyte depleted mice were infected IV with wildtype or Δ*fruR* strain. At 72 hours post-infection, mice were euthanized, and bacterial concentrations were determined in liver and spleen.

### Statistical analysis

The dot plots and median values of bacterial tissue concentrations in each mouse were generated using GraphPad Prism 8. For *in vivo* experiments, a non-parametric Mann-Whitney test was used to compare bacterial concentrations in liver and spleen of infected mice. Fold changes were calculated for each gene using 2^−ΔΔCt^ and used for statistical analysis to determine significant differences in gene expression between F2365Δ*fruR* and wildtype strain. Intracellular bacteria concentrations and plaque sizes were compared using a two-tailed Student’s *t* test. P value < 0.05 was considered significant.

## Results

### *fruR* contributes to *L*. *monocytogenes* virulence

In *L*. *monocytogenes*, *fruR* is located in the fructose operon with *fruA* and *fruB*, with *fruA* encoding fructose phosphotransferase and *fruB* encoding phosphofructokinase. The Δ*fruR* strain did not show growth defects in brain heart infusion (BHI) broth compared to wildtype strain F2365 (S2 Fig).

**Fig 1:**
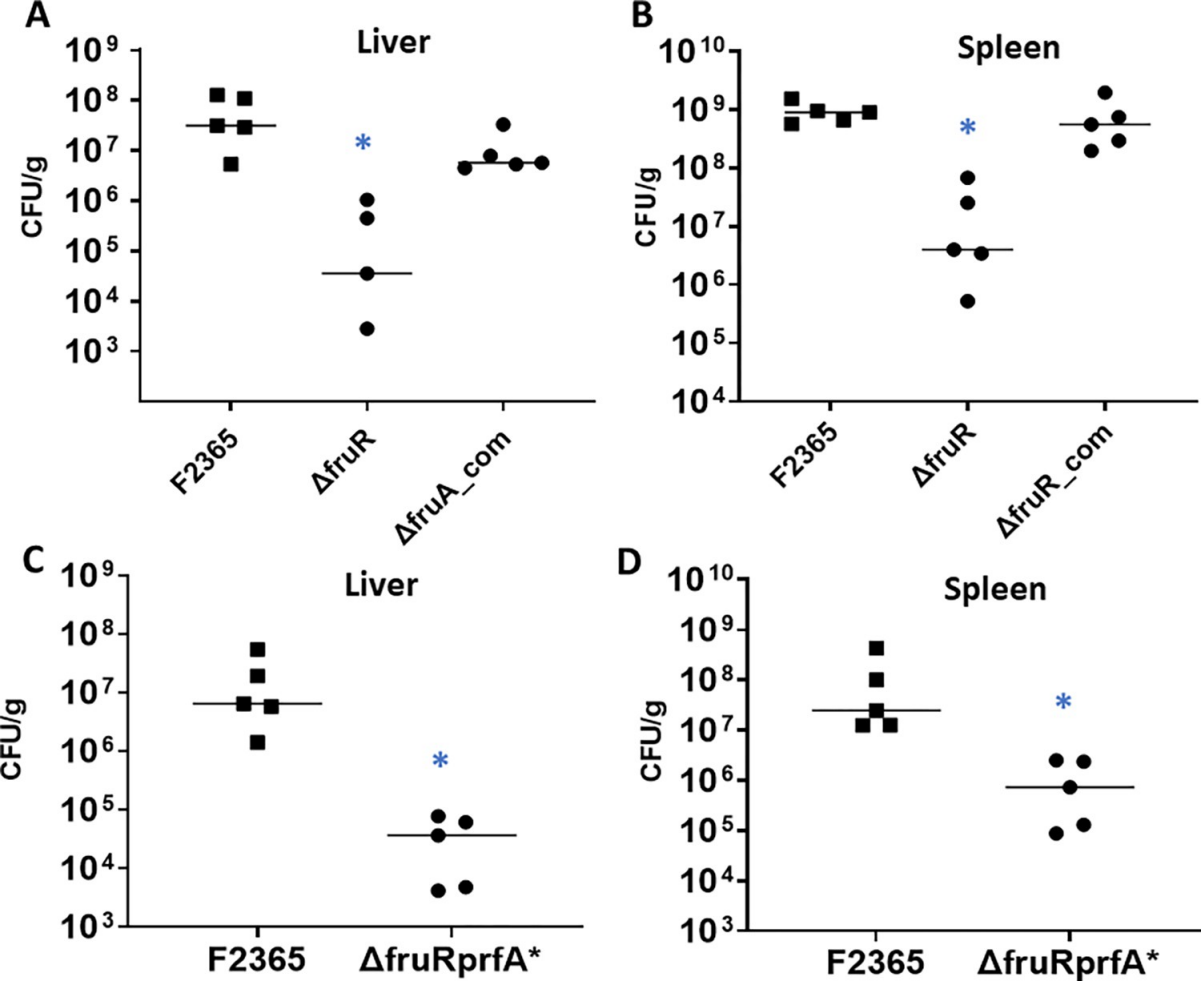
The Δ*fruR* strain is attenuated in mice at 72 hours post-infection and PrfA activation does not rescue attenuation of the Δ*fruR* strain. Each animal group (5 mice per cage) was IV infected with indicated strain. Livers (A and C) and spleens (B and D) were harvested at 72 hours post-infection and bacterial concentrations were determined. Data were analyzed using a nonparametric Mann-Whitney test. Median numbers for each strain are indicated by horizontal lines. Asterisks indicate significant differences (P* < *0.05) compared to the wildtype.

In 10–12-week-old female Balb/c mice, animals infected with Δ*fruR* exhibited 2.3 and 1.6 log_10_ decreased bacterial CFU at 72 hours post-infection in spleens and livers compared to animals infected with wildtype bacteria, respectively (Fig 1A and 1B). However, no significant difference in bacterial numbers were observed between Δ*fruR* and wildtype at 24-hour post-infection (S3 Fig). The difference in the Δ*fruR* strain clearance between early and late time points indicates that FruR is not required for early colonization or for the initial establishment of infection and suggests a possible role for FruR during the later stages of infection or in resistance to the innate immune responses. The complemented strain carrying a wildtype copy of *fruR* was able to restore bacterial concentrations to a level similar to the wildtype strain in the infected animals. Similar to IV infection, oral infection of BALB/c mice with Δ*fruR* resulted in lower bacterial concentrations in the liver and spleen at 5 days post-infection (S3 Fig).

### Constitutive activation of PrfA (PrfA*) does not restore virulence to Δ*fruR*

PrfA regulates expression of *L*. *monocytogenes* virulence genes and is essential for virulence [12,52]. We tested the hypothesis that the virulence defect in the Δ*fruR* strain is due to FruR directly affecting PrfA expression and/or activity. At 72 hours post-infection, bacterial concentrations in livers and spleens of mice infected with ΔfruRprfA* were significantly lower than wildtype (Fig 1C and 1D). This result indicates that the constitutive expression of PrfA* did not restore virulence to Δ*fruR* strain.

### Δ*fruR* is defective in cell-to-cell spread

**Fig 2:**
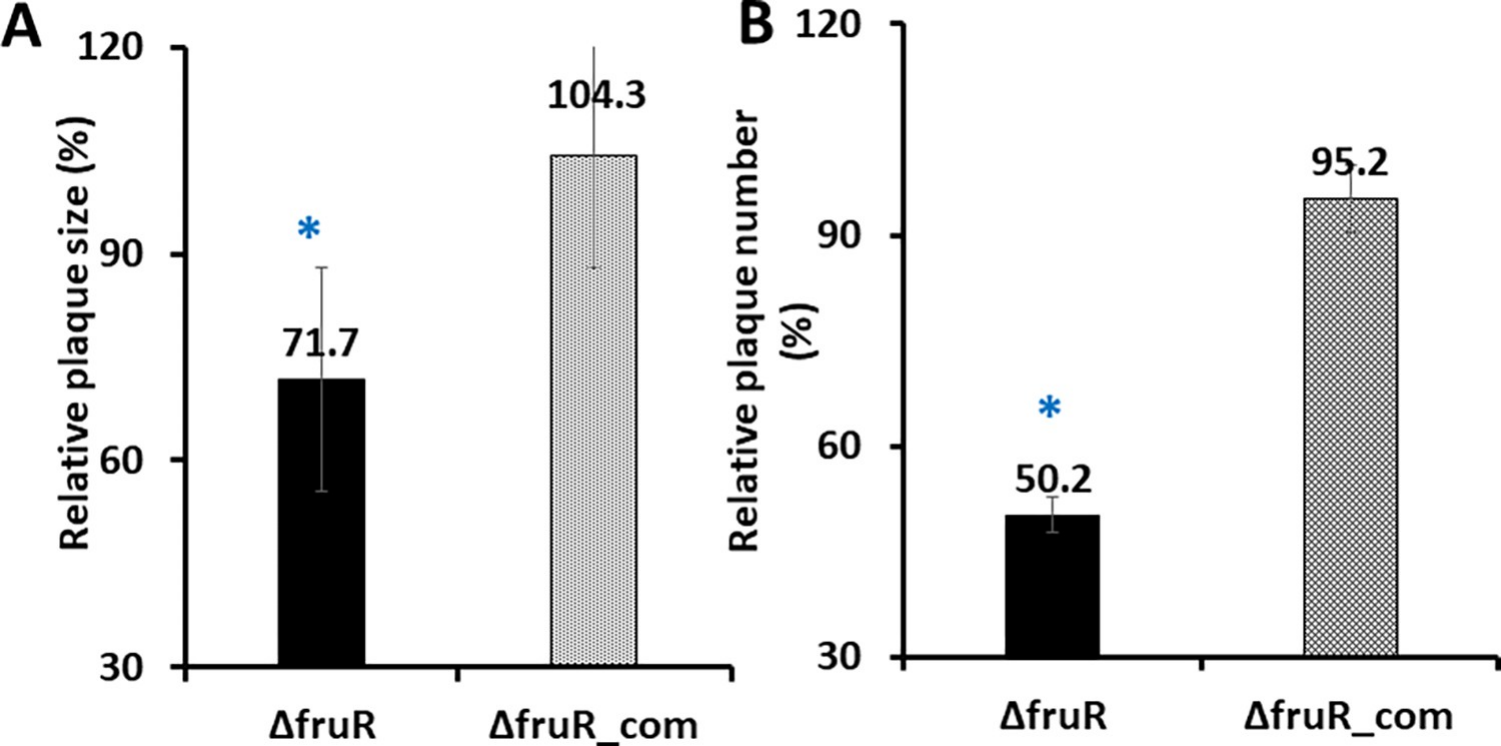
The Δ*fruR* strain has decreased plaque size and numbers in L2 fibroblast monolayers compared to wildtype and complemented Δ*fruR* (Δ*fruR_*com). (A) Relative plaque size is based on the percent plaque size compared to wildtype. (B) Relative plaque number is based on percent plaque number compared to wildtype. Monolayers of L2 fibroblasts were infected with the indicated strains, and plaque number and size were determined at 96 hours post infection. The experiment was repeated 3 independent times. At least 10 plaques were measured each time. Error bars represent the standard errors of the means and asterisks (*) indicate statistical significance (P < 0.05) by two-tailed Student’s *t* test compared to wildtype.

The ability of *L*. *monocytogenes* to form zones of clearing or plaques in murine L2 fibroblast cells is an indication of both bacterial escape from host cell vacuoles as well as bacterial cell-to-cell spread [53]. We therefore examined the ability of Δ*fruR* mutants to form visible plaques in monolayers of L2 fibroblasts (Fig 2). Mutants lacking *fruR* exhibited fewer numbers of plaques (an average of 49.8% reduction in plaque numbers) and the plaques formed were smaller in size (28.3% reduction in plaque size) in comparison to cell infected with wildtype (Fig 2A and 2B). These findings indicate a significant contribution of FruR to cellular invasion and suggest an additional defect exists that reduces intracellular replication and/or cell-to-cell spread.

### Contribution of FruR to intracellular replication

**Fig 3:**
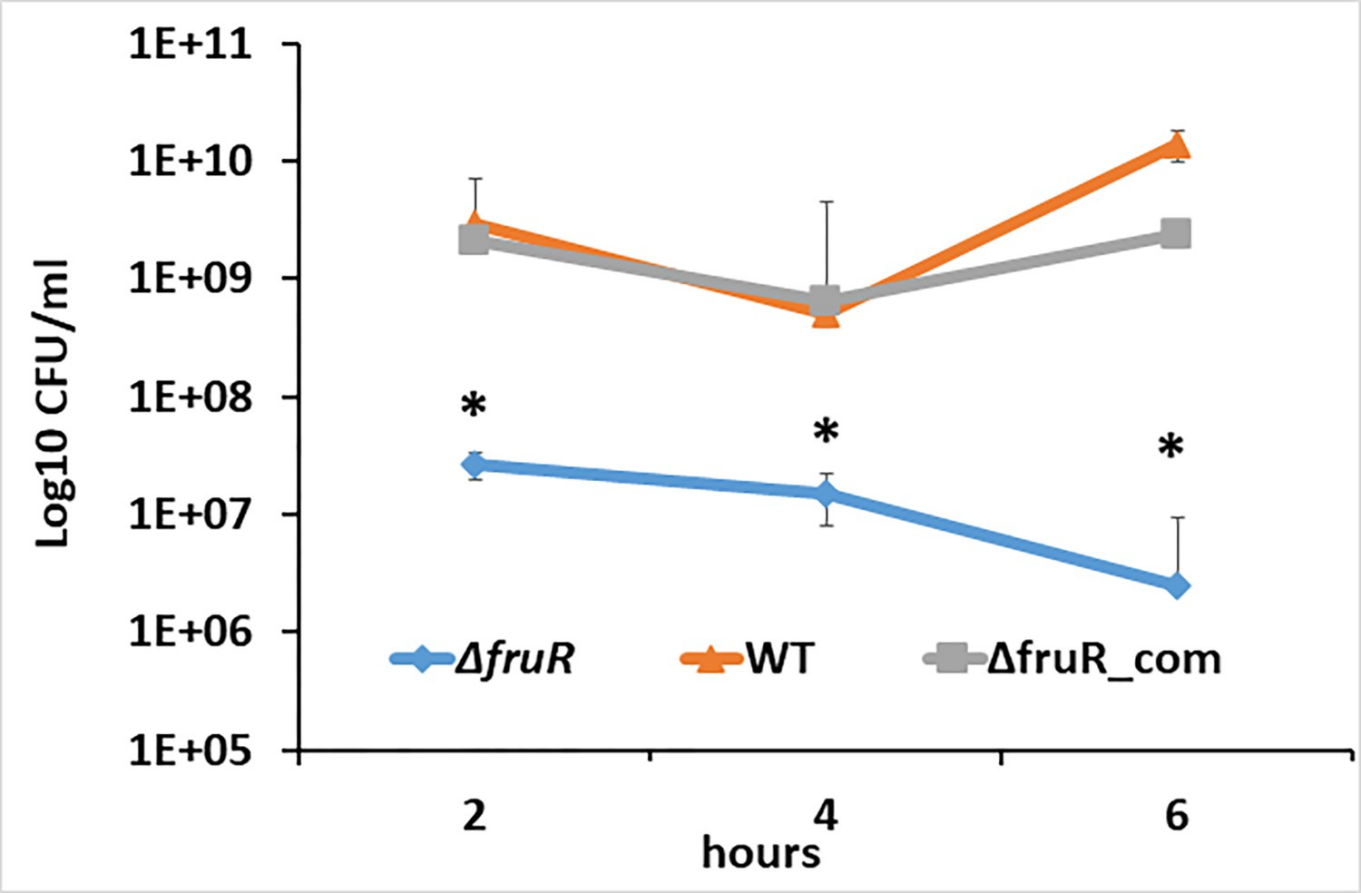
Intracellular replication of wildtype *L*. *monocytogenes* and Δ*fruR*. J774A.1 macrophage cells were infected with Δ*fruR*, wildtype (WT), and complemented Δ*fruR* (Δ*fruR*_com). After infection, monolayers were washed, and medium containing gentamicin was added. At 2-, 4-, and 6-hours post-infection, macrophages were lysed, and released intracellular bacteria were quantified by determining CFU/ml in the lysate on BHI agar plates. All infections were performed in five replicates, and two independent assays were performed. The statistical significance between Δ*fru*R and wildtype at each indicated time point was determined using a student *t* test. Asterisks indicate statistical significance, with P < 0.05.

J774A.1 macrophage cells were used to assess the intracellular replication of the Δ*fruR* strain in comparison to cells infected with wildtype [54]. We found that intracellular numbers of Δ*fruR* were significantly decreased (P < 0.05) compared to wildtype F2365 at 2-, 4-, and 6-hours post-infection (Fig 3). However, the pattern of intracellular growth for Δ*fruR* was indistinguishable from wildtype. Therefore, while the plaque assay points to a defect in intracellular replication and/or cell-to-cell spread, this assay suggests that intracellular replication occurs, and thus the plaque assay results may be due to a defect in cell-to-cell spread.

### Δ*fruR* has decreased growth rate in defined media supplemented with glucose

**Fig 4:**
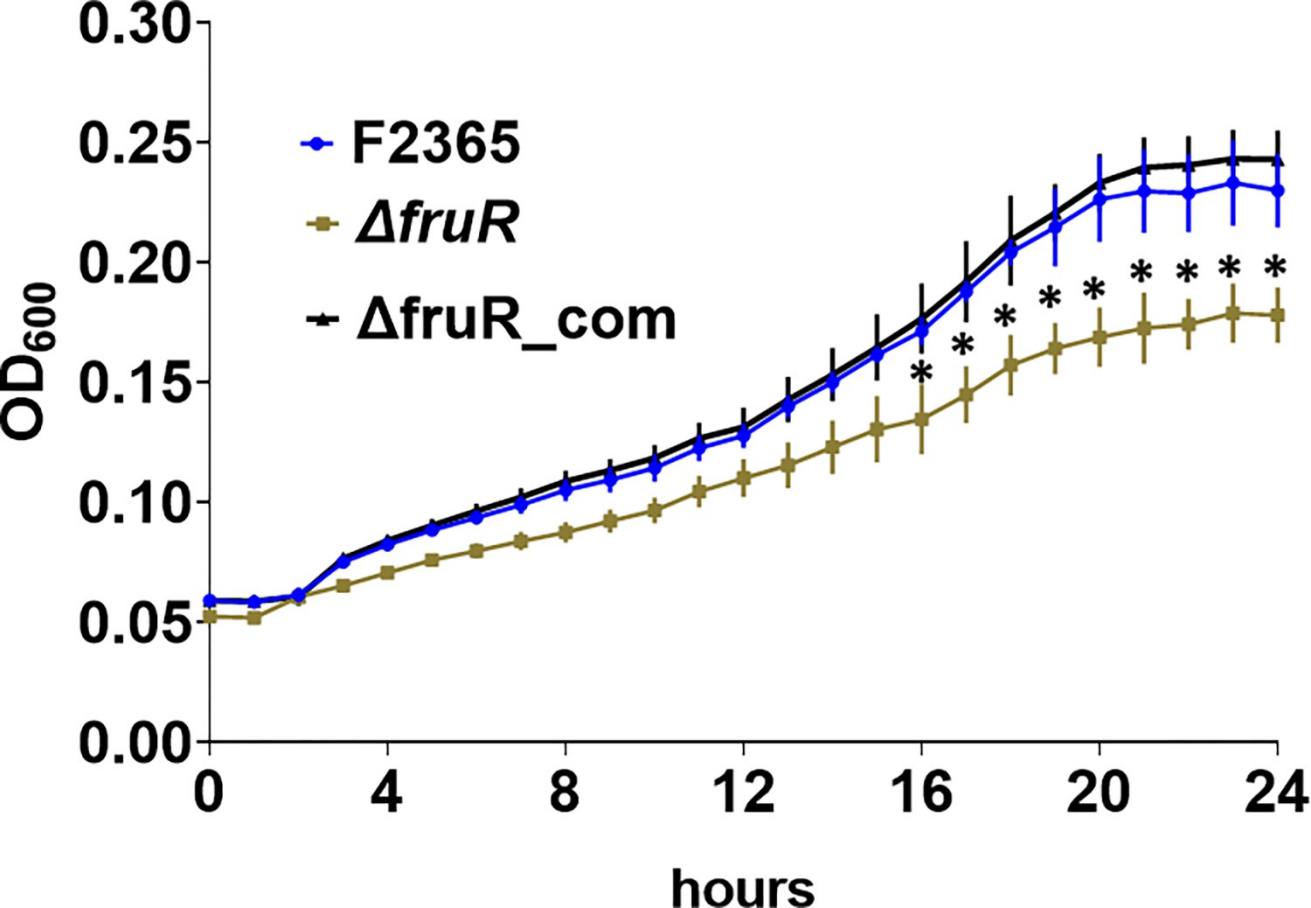
Growth curves of wildtype *L*. *monocytogenes* F2365 and Δ*fruR* in minimal medium with 50 mM glucose. Growth assays were performed in a 24-well plate, and data are averages of three independent experiments with four replicates each.

To determine whether FruR facilitates uptake and utilization of carbon sources, we compared growth of Δ*fruR* with F2365 and complemented strain in minimal medium (MM) supplemented with different carbon sources. After 16 hours, growth of Δ*fruR* was significantly lower than the growth of wildtype in MM with glucose (Fig 4). In contrast, Δ*fruR* did not have decreased growth in MM supplemented with fructose, mannose, maltose, or sucrose (S4 Fig). This result suggests that FruR plays a specific role in glucose uptake and/or metabolism during stationary growth phase.

### Glycolysis and pentose phosphate pathway (PPP) genes are regulated by *fruR*

To determine the impact of FruR on *L*. *monocytogenes* gene expression, RNA-seq was used to compare Δ*fruR* to wildtype grown in BHI. Results of genes with false discovery rate <0.05 are shown in Table 2. In total, 12 genes were upregulated (threshold >1.5-fold), while 16 genes were downregulated in Δ*fruR*. In particular, genes encoding pentose phosphate pathway (PPP) enzymes and enzymes for NADPH synthesis were decreased in the Δ*fruR* strain, including glucose 6-phosphate dehydrogenase (*zwf*), 6-phosphogluconate dehydrogenase (*gnd)*, transketolase (*tkt*), and transaldolase (*taldo*). Moreover, Δ*fruR* had upregulated genes encoding glycolysis enzymes, such as phosphofructokinase (*fruB*) and glucokinase *(glck)* [55]. These findings suggest that **(i)** FruR controls *L*. *monocytogenes* carbon metabolism by regulating the expression of genes encoding glycolysis and PPP enzymes, and **(ii)** FruR induces expression of genes encoding PPP enzymes and suppresses glycolysis genes.

**Table 2:** Genes with significantly changed expression in Δ*fruR* strain.

	Locus Tag	Protein name	Fold changes
**Upregulated**			
	LMOf2365_2305	PTS fructose transporter subunit IIC (FruA)	8.30
	LMOf2365_2306	1-phosphofructokinase (FruB)	9.35
	LMOf2365_0272	hypothetical protein	1.56
	LMOf2365_0568	tagatose 1,6-diphosphate aldolase	1.77
	LMOf2365_0604	GntR family transcriptional regulator	1.73
	LMOf2365_0588	Magnesium transporter CorA family protein	1.56
	LMOf2365_0817	Hypothetical protein	1.92
	LMOf2365_1015	DUF4064 domain-containing protein	1.50
	LMOf2365_1473	RNA polymerase sigma factor RpoD	1.87
	LMOf2365_1718	TIGR01777 family protein	1.69
	LMOf2365_2246	Antibiotic biosynthesis monooxygenase	1.51
	LMOf2365_2391	Hypothetical protein	1.61
**Downregulated**			
PPP	LMOf2365_1356	Glucokinase (*glcK*)	-5.97
	LMOf2365_2002	Glucose 6-phosphate dehydrogenase (*zwf*)	-1.81
	LMOF2365_1395	6-phosphogluconate dehydrogenase (*gnd*)	-1.64
	LMOf2365_0529	Transaldolase (*taldo 1*)	-2.84
	LMOf2365_2692	Gluconokinase (*gntK*)	-3.97
	LMOf2365_1053	Transketolase (*tkt-1*)	-1.71
	LMOf2365_0057	Putative two-component sensor histidine	-1.45
	LMOf2365_0508	putative accessory gene regulator protein D	-2.19
σ^B^ regulator	LMOf2365_0908	rsbT co-antagonist protein RsbR (*rsbR*)	-1.68
	LMOf2365_0909	anti-sigma factor B antagonist (*rsbS*)	-1.86
	LMOf2365_0910	anti-sigma B factor RsbT (*rsbT*)	-1.63
	LMOf2365_0911	sigma factor B regulator protein (RsbU)	-1.72
	LMOf2365_1334	1-deoxy-D-xylulose 5-phosphate reductoisomerase (dxr)	-1.86
	LMOf2365_1437	Type I phosphodiesterase/nucleotide pyrophosphatase family protein	-1.82
	LMOf2365_2119	MATE efflux family protein	-1.51
	LMOf2365_2272	Acetoin utilization transport system permease protein	-1.67

In addition, four different regulators of sigma B factor (*rsbR*, *rsbS*, *rsbT*, and *rsbU*) were downregulated in Δ*fruR*. These genes are in the *sigB* operon of *L*. *monocytogenes* and contribute to regulation of σ^B^ activity in response to environmental stresses and energy stresses [56]. RNA-seq results were confirmed using qRT-PCR and primers for *fruA*, *fruB*, *glck*, and *rsbT* genes (S5 Fig).

### FruR is required for *L*. *monocytogenes* response to oxidative stress

**Fig 5:**
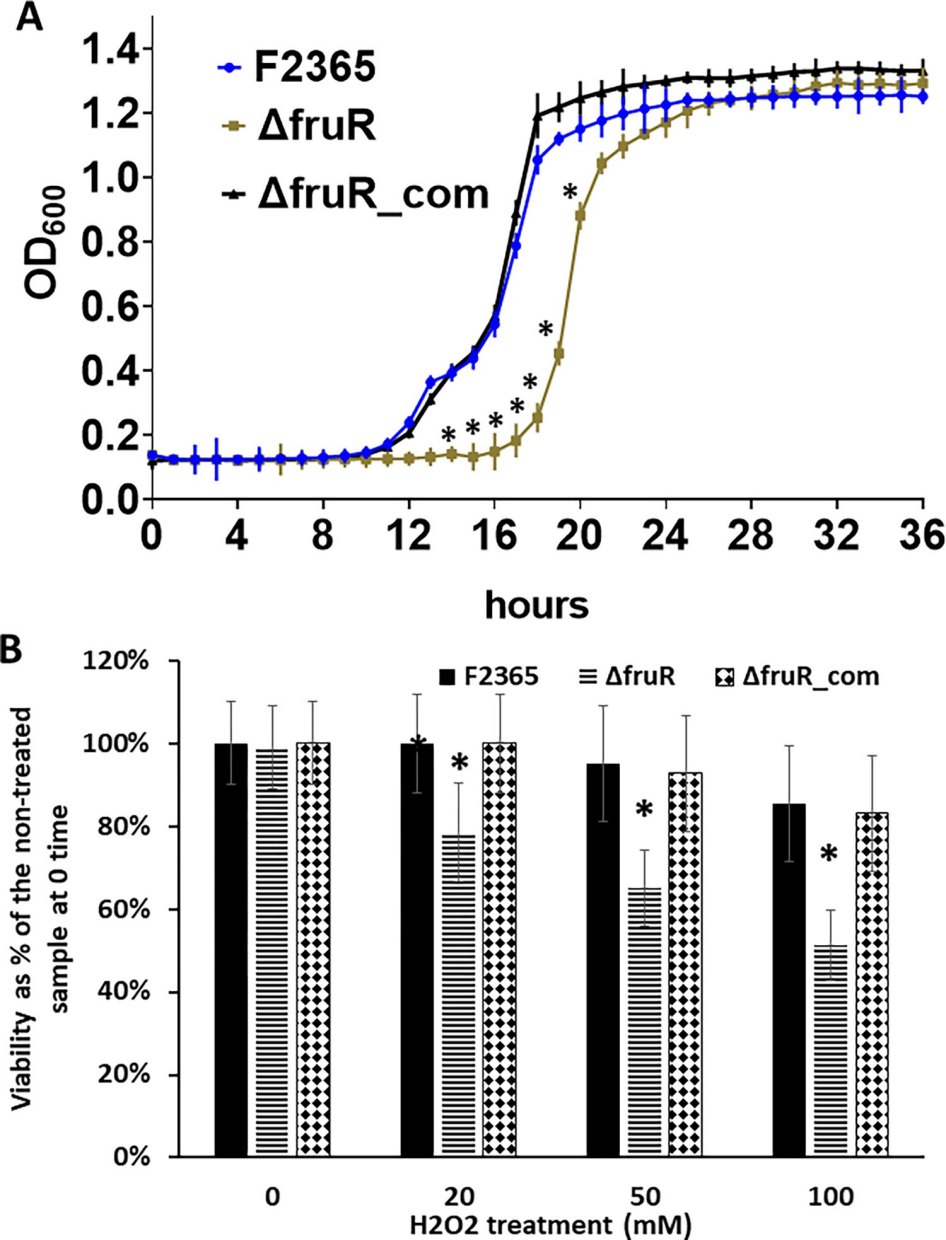
FruR is required for *L*. *monocytogenes* response to oxidative stress. (A) The Δ*fruR* strain has increased lag phase compared to wildtype during growth in BHI broth with 10 mM H_2_O_2_. Growth assays were performed in a 24-well plate and were repeated three independent times with five replicates. Error bars represent SEM. Asterisks mark time points at which OD_600_ were significantly different (P < 0.05). (B) Survival of Δ*fruR*, wildtype (F2365), and complemented (Δ*fruR*_com) strains after exposure to H_2_O_2_. *L*. *monocytogenes* strains were grown to mid-log phase, resuspended in BHI, and treated with H_2_O_2_. After 1 hour, CFU were enumerated by colony counts on BHI agar plates. Percent survival was calculated by dividing bacterial concentration (CFU/ml) after treatment with H_2_O_2_ by the initial CFU/ml for the same strain prior to treatment (time point 0). Data are the mean ± SEM of three experiments with five replicates. At each concentration, Student t-test was used to compare the survival percent. Asterisks indicate significant differences (P < 0.05) compared to the wildtype.

Based on RNA-seq findings, we hypothesized that Δ*fruR* strain might be more sensitive to oxidative stress due to defective NADPH production via PPP. Exposure to H_2_O_2_ is widely used to study oxidative stress in bacteria [57,58]. First, we compared growth in BHI medium with 10 mM H_2_O_2_, and we found that Δ*fruR* had an increased lag phase compared to wildtype (Fig 5A). By comparison, Δ*fruR* did not show growth defects in BHI broth (without H_2_O_2_) relative to wildtype.

Next, we compared survival of wildtype, Δ*fruR*, and complemented strain after exposure to H_2_O_2_ for 1 hour in BHI. At 20, 50, and 100 mM of H_2_O_2_, wildtype strain viability was significantly higher than viability of Δ*fruR* after exposure for 1 hour, indicating that FruR contributes to survival of *L*. *monocytogenes* during oxidative stress (Fig 5B). These results suggest a novel virulence mechanism whereby FruR upregulates the PPP in *L*. *monocytogenes*, which is necessary for full protection from oxidative stress.

### FruR contributes to *L*. *monocytogenes* resistance to neutrophil/monocyte-mediated killing in mice

**Fig 6:**
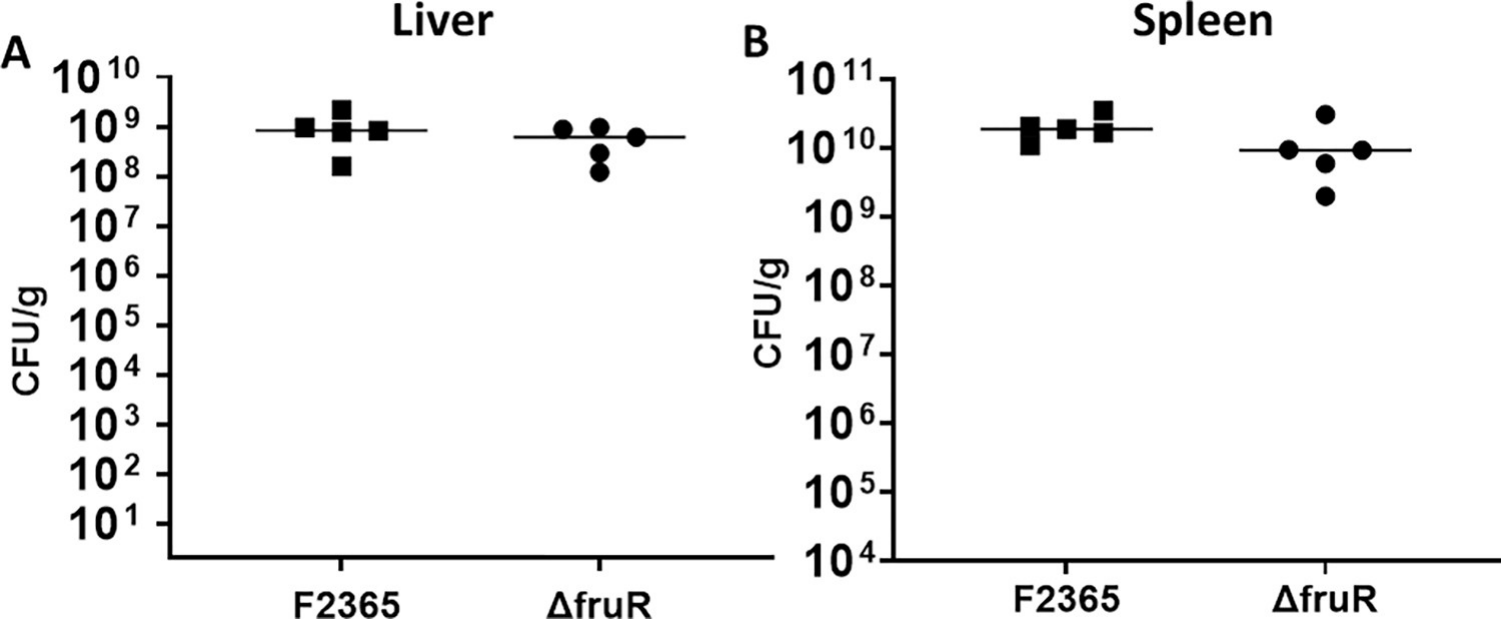
The contribution of FruR to neutrophil/monocytes resistance. The Δ*fruR* strain has similar virulence as wildtype strain in mice depleted of neutrophils/monocytes. Mice (5 mice per cage) were injected IV with monoclonal antibody RB6 at 24 and 6 hours before experimental infection. Livers and spleens were harvested at 72 hours post-infection. There was no significant difference in CFU observed between Δ*fruR* strain and wildtype.

We investigated whether attenuation of Δ*fruR* could be caused by increased susceptibility to innate immune cells. For this experiment, we used monoclonal antibody RB6 to deplete both neutrophils and inflammatory monocytes from mice [59]. The specificity and efficacy of RB6 is well established [60]. Following neutrophil/monocyte depletion, Δ*fruR* had similar virulence to wildtype in mice at 72 hours post-infection (Fig 6). These results suggest that the virulence attenuation of Δ*fruR* is neutrophil/monocyte-dependent and supports the new hypothesis that FruR has a specific function in protecting *L*. *monocytogenes* from neutrophil/monocyte-mediated killing.

## Discussion

FruR is annotated as a DeoR-family transcriptional regulator. *L*. *monocytogenes* FruR comprises 250 amino acids and includes a helix-turn-helix DNA-binding motif at the N-terminal region and a sensor domain at the C-terminal region. A previous study has shown significant upregulation of a gene encoding a DeoR-family protein (LMOF6854_2396, paralogous to FruR), when comparing transcriptomic sequencing of persistent and nonpersistent *L*. *monocytogenes* EGD-e strains grown in the presence of quaternary ammonium compounds (QACs) [61]. In another study, a DeoR-family transcriptional regulator gene (paralogous to FruR) was downregulated after 4 hours of exposure of *L*. *monocytogenes* to carnocyclin A, an antimicrobial peptide used to control the growth of *L*. *monocytogenes* in meat products [62]. Thus, it is probable that DeoR-family proteins, including FruR, have complex regulatory roles in *L*. *monocytogenes*, and their functions appear to include several cellular processes. The current study was undertaken to understand the importance of FruR in *L*. *monocytogenes* pathogenesis.

Our results indicate, for the first time, that FruR significantly contributes to *in vitro* and *in vivo* pathogenesis, highlighted by attenuation in mice following IV injection and impaired cell-to-cell spread in fibroblast cells. This finding is corroborated by previous studies that have connected DeoR-family regulators with bacterial pathogenesis. For example, FruR of *Streptococcus pyogenes* is important for survival in whole human blood and resistance to killing by human neutrophils and primary monocytes. However, FruR did not contribute to virulence in a murine model of infection, perhaps relevant to its replication niche (*S*. *pyogenes* is often found extracellular) [45,63]. In addition, a *Brucella suis deoR1* mutant was shown to be strongly attenuated in human macrophages [64].

Previous studies demonstrated an existence link between regulation of utilizable carbon sources and expression of virulence genes in *L*. *monocytogenes* [35,65]. Given the role of DeoR family of transcriptional regulators in sugar metabolism, we asked the question whether the attenuation of Δ*fruR* strain could be explained by reduction in the PrfA activity. The introduction of the prfA* mutation to the *fruR* strain did not restore the virulence of Δ*fru*R strain, demonstrating that FruR has no regulatory role or connection with PrfA activity and suggesting that the Δ*fruR* colonization defect is due to a mechanism distinct from *L*. *monocytogenes* PrfA activation.

To provide further insight into the regulatory role of FruR in *L*. *monocytogenes*, we identified the genes controlled by FruR using RNA-seq. Of particular interest is that FruR functions to induce the expression of genes encoding PPP enzymes and to suppress glycolysis. Deletion of *fruR* downregulated genes encoding two PPP enzymes, glucose 6-phosphate dehydrogenase (*zwf*) and 6-phosphogluconate dehydrogenase (*gnd*). Zwf catalyzes conversion of glucose-6-phosphate (G6P) to 6-phosphogluconolactone (6PGL), generating NADPH from NADP. Gnd converts 6PGL into ribulose-5-phosphate (Ru5P), generating a second molecule of NADPH (S6 Fig). The PPP plays a crucial role in maintaining redox homeostasis in bacteria [66]. In *L*. *monocytogenes*, NADPH is used as the reducing agent for numerous reductase mechanisms involved in detoxification and protection from reactive oxygen species [14,67–69]. Therefore, our findings may indicate that a functional FruR is important for *L*. *monocytogenes* pathogenesis to regenerate sufficient NADPH to resist oxidative stress by directing carbon flow from glycolysis to the PPP pathway.

We therefore asked whether FruR contributes to *L*. *monocytogenes* resistance to oxidative stress using hydrogen peroxide (H_2_O_2_), which is widely used to study oxidative stress [70,71]. The Δ*fruR* mutant had a significant growth defect and reduced viability as result of exposure to H_2_O_2_. This result correlates with our RNA-seq findings showing that FruR induces the PPP, which is needed for NADPH production. Redirection of metabolic flux from glycolysis to the PPP provides protection against oxidative stress in yeast and mammalian cells, but this has not been thoroughly examined in prokaryotes [72–76]. Future work will explore the role of FruR in the induction of this shift and appropriately adjusting the *L*. *monocytogenes* response to oxidative stress. We will also explore the role of NADPH production enzymes in oxidative stress response.

Transcriptome analysis of Δ*fruR* also revealed downregulation of four genes encoding regulators of the *sigB* operon: *rsbR*, *rsbS*, *rsbT*, and *rsbU* (Table 2). In *L*. *monocytogenes*, the *sigB* operon encodes seven regulators of sigma B: RsbR, RsbS, RsbT, RsbU, RsbV, RsbW, and RsbX [56,77]. Bacterial σ^B^ plays an important role in coordinating gene expression in response to various environmental stresses, including high osmolarity, low pH, cold exposure, and antimicrobial stress [78]. In addition to its stress-protective function, σ^B^ regulates many other *L*. *monocytogenes* processes, including biofilm formation, cell-wall turnover, survival within the mammalian gastrointestinal (GI) tract, and regulation of carbon and energy metabolism [16,79]. It is possible that downregulation of σ^B^ in Δ*fruR* contributes to its attenuation and its survival defect in an oxidative environment. Thus, FruR may contribute to induction of σ^B^ activity under stress conditions. However, further studies are necessary to determine the nature of the interaction between FruR and σ^B^ factor.

Oxidative stress damage generated by production of reactive oxygen species (ROS) (such as superoxide radicals, hydrogen peroxide, hypochlorous acid, and hydroxyl radicals) is a potent bactericidal mechanism by which professional phagocytes (neutrophils, monocytes, and macrophages) limit *L*. *monocytogenes* systemic spread [80,81]. Therefore, we tested the hypothesis that FruR protects *L*. *monocytogenes* from oxidative stress imposed by neutrophils and monocytes. To this end, neutrophils/monocytes were depleted in mice with the monoclonal antibody RB6-8C5 prior to infection with wildtype and Δ*fruR* strains. At 72 hours after infection, Δ*fruR* exhibited a similar level of virulence compared to wildtype as indicated by bacterial concentrations in the liver and spleen of neutrophil-depleted mice. This suggests that neutrophils/monocytes play a significant role in the attenuation of Δ*fruR* compared to wildtype. It was reported that FruR is important for *Streptococcus pyogenes* survival in whole human blood and resistance to killing by human neutrophils and primary monocytes [82]. However, the molecular mechanism by which FruR mediates resistance to neutrophils was not determined. Our results showed that Δ*fruR* strain failed to upregulate PPP, and Δ*fruR* is more susceptible to oxidative killing. Because oxygen radicals are an important killing mechanism used by phagocytes, it is logical that generation of NADPH via the PPP is important for listerial resistance to phagocytes. Thus, we propose a new hypothesis whereby FruR mediates resistance of *L*. *monocytogenes* to phagocytes and oxidative stress by upregulation of PPP and increased NADPH production. Further, our results provide evidence that regulation of carbon flux from glycolysis to PPP plays a significant role in the pathogenesis of *L*. *monocytogenes*.

Reduced growth of Δ*fruR* in defined medium with glucose as the only carbon source was another interesting finding in our study. The Δ*fruR* strain did not show any growth defects in BHI, a nutrient-rich media. The defined medium contains limited resources and nutrients compared to nutrient-rich BHI. This result suggests that FruR contribute to glucose uptake in a nutrient-limited environment. FruR belongs to the DeoR family of transcriptional regulators, which in most instances act as transcriptional repressors in sugar metabolism [83]. Our transcriptome comparison between wildtype and Δ*fruR* revealed that FruR functions as a negative regulator for PTS fructose transporter subunit IIC (FruA) and 1-phosphofructokinase (FruB). Repression of FruA and FruB by FruR has been observed in other bacterial species [20,63]. The growth defect of Δ*fruR* in MM with glucose may be due to the upregulation of glucokinase *(glck)*, which encodes an enzyme that catalyzes phosphorylation of glucose to glucose-6-phosphate (G6P) [55].

In conclusion, we showed that FruR contributes to *L*. *monocytogenes* virulence, survival during oxidative stress, and resistance to neutrophils/monocytes. Moreover, we found that FruR of *L*. *monocytogenes* act as a transcriptional activator for PPP, which is an important pathway for generation of NADPH and protection from oxidative stress. Transcriptional analysis also showed that FruR induces the sigma B factor operon. Sigma B is important for stress adaptation and controls the response to several stress cues, such as osmotic stress, light, and acid stress. Overall, this work suggests a novel virulence mechanism in which FruR upregulates the PPP in *L*. *monocytogenes*, and this upregulation is necessary for full protection from oxidative stress and phagocytic killing. FruR is the first transcriptional regulator of the DeoR-type characterized to date in *L*. *monocytogenes*. Further understanding of the regulatory mechanisms of FruR in *L*. *monocytogenes* could provide important information on how the pathogen uses regulation of metabolic pathways to adapt to stressful environments, including host phagocytic killing mechanisms.

## Supporting information

S1 Fig A phylogenetic tree of the DeoR-family regulators in *L*. *monocytogenes* F2365 strain. The seven DeoR-family regulators are classified into two clades. In the first clade, the DeoR-family regulators are characterized by the presence of both DNA-binding domain at the N-terminus and sensor domain at the C-terminus. Six DeoR-family members, including FruR, are in the first clade. The second clade has one DeoR-family protein; it has the DNA-binding domain and sugar binding domain. The phylogenetic tree was constructed using the unweighted-pair group method with arithmetic mean. Multiple sequence alignment was conducted with CLUSTALW.
(TIF)

S2 Fig The Δ*fruR* strain did not show growth defects in BHI, as enriched medium. The growth assay was conducted with *L*. *monocytogenes* strain F2365, Δ*fruR*, and complement strain. The growth kinetic was monitored using optical density measurements at 600 nm. The experiment was repeated three independent times with four replicates and the figure shows a representative experiment. Error bars represent SEM. The optical density values between Δ*fruR* strain and wildtype were not statistically significant (P > 0.05).
(TIF)

S3 Fig Bacterial counts (CFU/g) of *L*. *monocytogenes* strain F2365, Δ*fruR* strain, and complemented strain following IV and oral infection in Balb/c mice. (A and B) Bacterial concentrations in livers and spleens upon IV infection via the tail vein with 2 x10^4^ CFU and dissected at 24 hours post-infection. Differences in bacterial concentrations between Δ*fruR* strain and wildtype were not statistically significant (P > 0.05). (C and D) Bacterial concentrations in livers and spleens upon oral infection by gavage needle with 5.5x10^6^ CFU and dissected at 5 days post-infection. Data were analyzed using a nonparametric Mann-Whitney test. Median numbers for each strain are indicated by horizontal lines. Asterisks indicate significant differences (P* < *0.05) compared to the wildtype.
(TIF)

S4 Fig Δ*fruR* strain exhibits normal growth patterns in MM supplemented with fructose (A), mannose (B), maltose (C), or sucrose (D) as a sole carbon source. Bacterial growth curves were determined by optical density measurements at 600 nm. All growth data are the results from three independent experiments with four replicates and the figure shows a representative experiment. Error bars represent SEM. Differences between Δ*fruR* strain and wildtype were not statistically significant (P > 0.05).
(TIF)

S5 Fig RT-PCR analyses of upregulated genes (*fruA* and *fruB*) and downregulated genes (*glck* and *rsbT*) in Δ*fruR* compared to wildtype. The data represent means ± standard errors from three biological replicates.
(TIF)

S6 Fig Simplified diagram of glycolysis and PPP in *L*. *monocytogenes*. (TIF)
