# Supplementary figures and images for: Role of FruR transcriptional regulator in virulence of Listeria monocytogenes and identification of its regulon
Source: PLoS One. 2022 Sep 2;17(9):e0274005. doi: 10.1371/journal.pone.0274005 (PMC9439231; doi:10.1371/journal.pone.0274005)

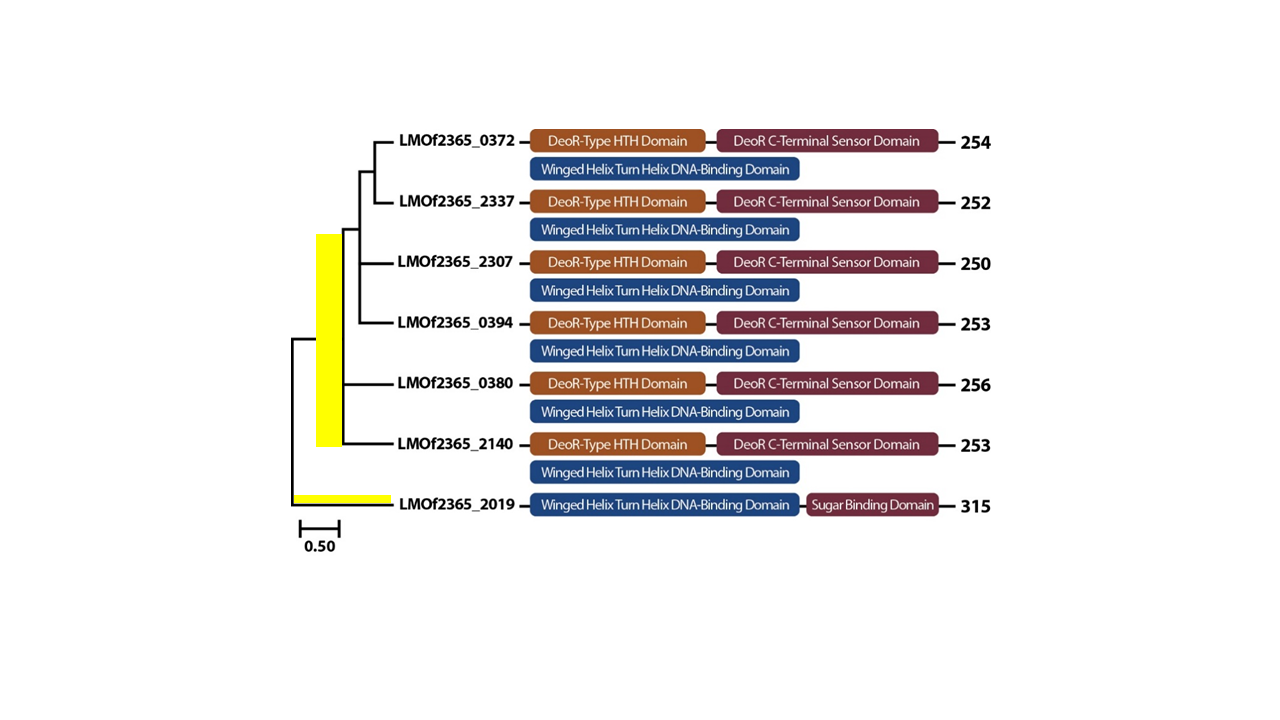

Supplement: S1 Fig — The seven DeoR-family regulators are classified into two clades. In the first clade, the DeoR-family regulators are characterized by the presence of both DNA-binding domain at the N-terminus and sensor domain at the C-terminus. Six DeoR-family members, including FruR, are in the first clade. The second clade has one DeoR-family protein; it has the DNA-binding domain and sugar binding domain. The phylogenetic tree was constructed using the unweighted-pair group method with arithmetic mean. Multiple sequence alignment was conducted with CLUSTALW. (TIF) [file pone.0274005.s001.tif]

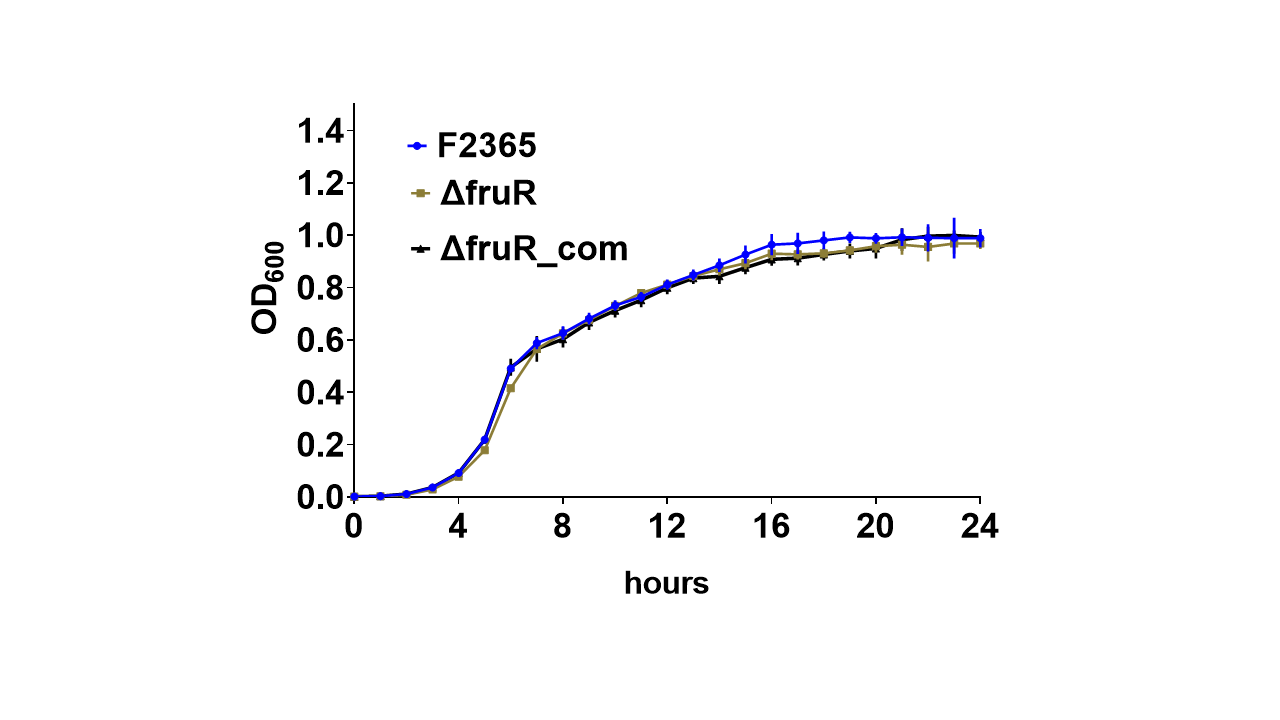

Supplement: S2 Fig — The growth assay was conducted with L. monocytogenes strain F2365, ΔfruR, and complement strain. The growth kinetic was monitored using optical density measurements at 600 nm. The experiment was repeated three independent times with four replicates and the figure shows a representative experiment. Error bars represent SEM. The optical density values between ΔfruR strain and wildtype were not statistically significant (P > 0.05). (TIF) [file pone.0274005.s002.tif]

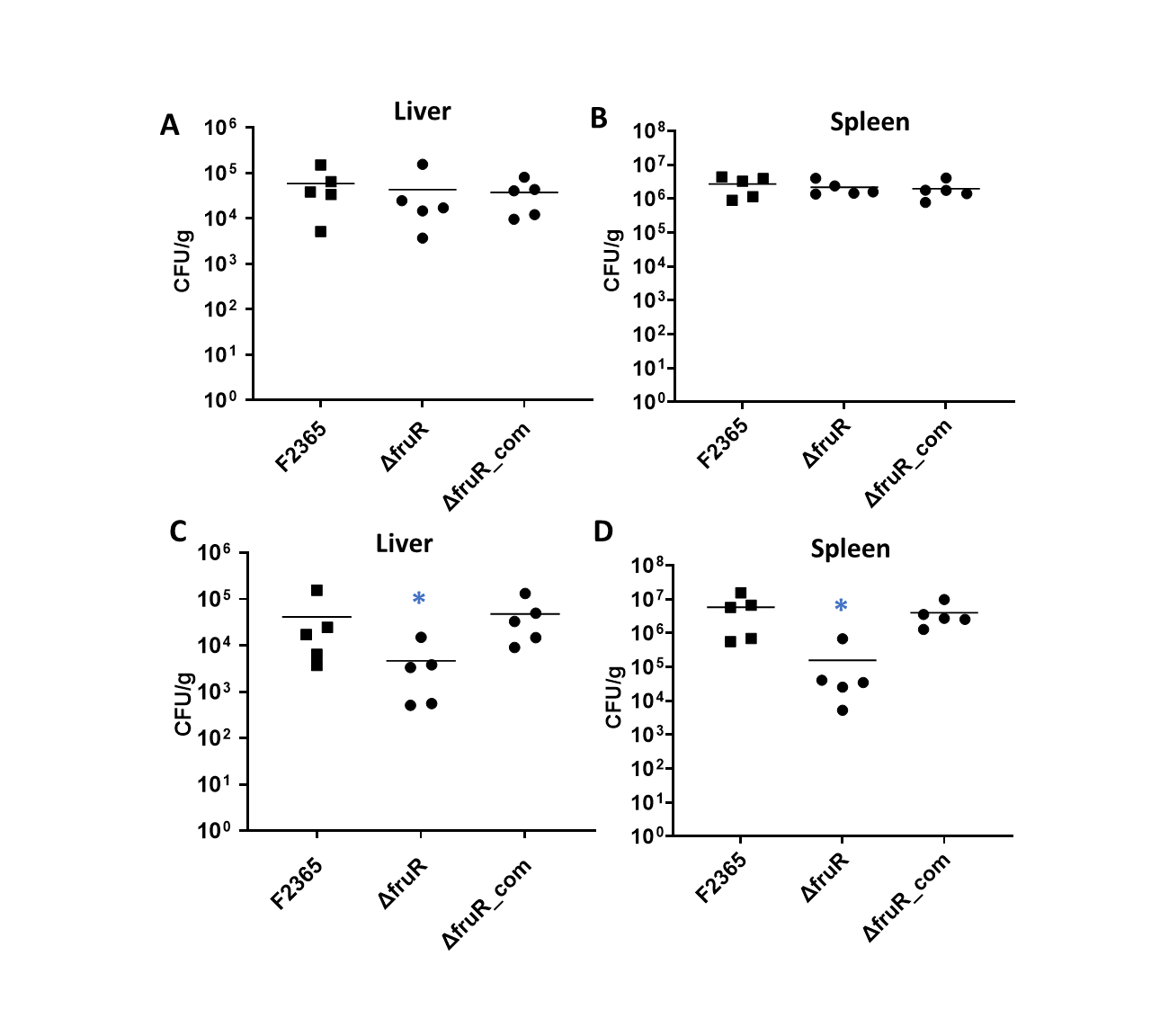

Supplement: S3 Fig — (A and B) Bacterial concentrations in livers and spleens upon IV infection via the tail vein with 2 x10^4^ CFU and dissected at 24 hours post-infection. Differences in bacterial concentrations between ΔfruR strain and wildtype were not statistically significant (P > 0.05). (C and D) Bacterial concentrations in livers and spleens upon oral infection by gavage needle with 5.5x10^6^ CFU and dissected at 5 days post-infection. Data were analyzed using a nonparametric Mann-Whitney test. Median numbers for each strain are indicated by horizontal lines. Asterisks indicate significant differences (P < 0.05) compared to the wildtype. (TIF) [file pone.0274005.s003.tif]

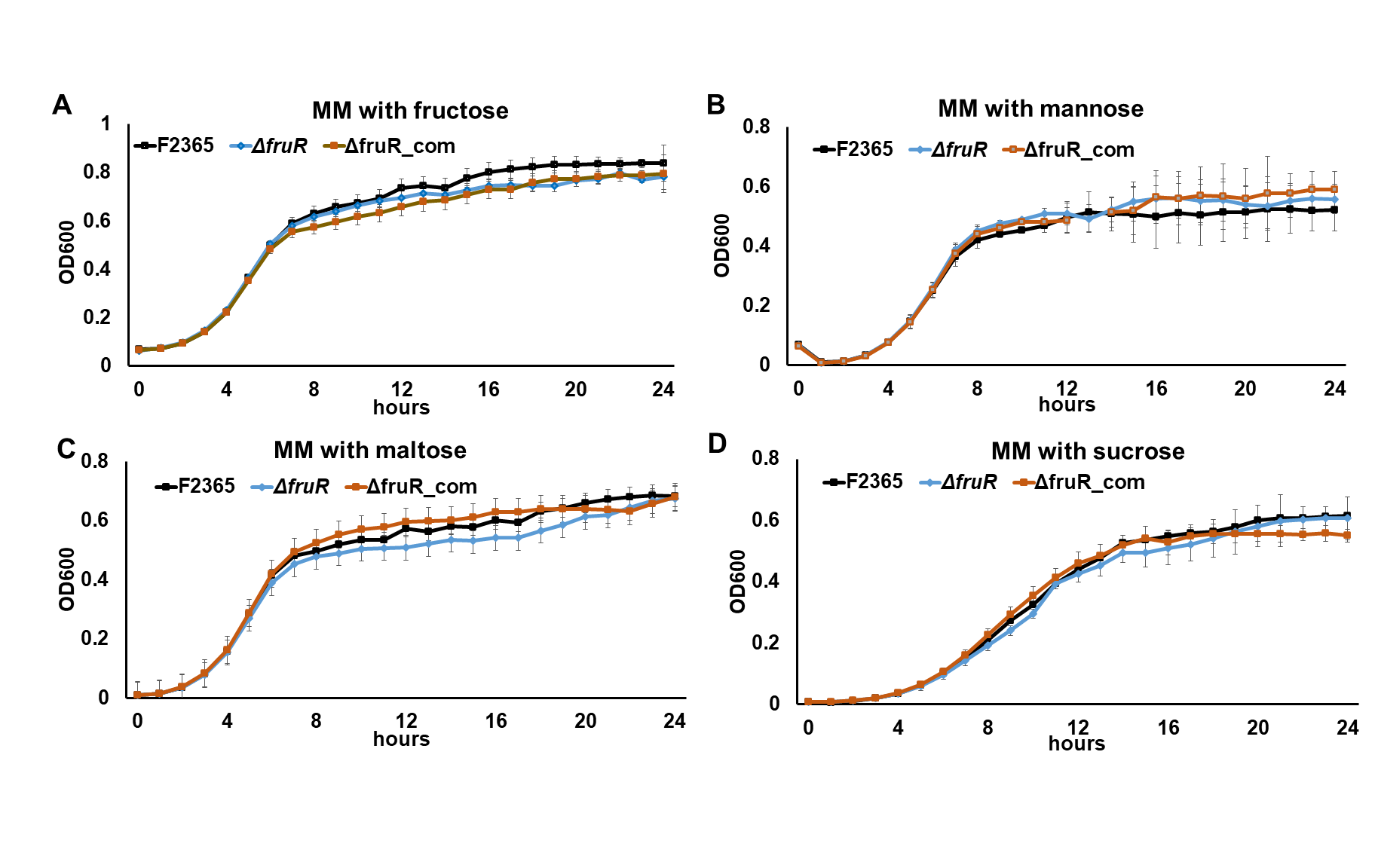

Supplement: S4 Fig — ΔfruR strain exhibits normal growth patterns in MM supplemented with fructose (A), mannose (B), maltose (C), or sucrose (D) as a sole carbon source. Bacterial growth curves were determined by optical density measurements at 600 nm. All growth data are the results from three independent experiments with four replicates and the figure shows a representative experiment. Error bars represent SEM. Differences between ΔfruR strain and wildtype were not statistically significant (P > 0.05). (TIF) [file pone.0274005.s004.tif]

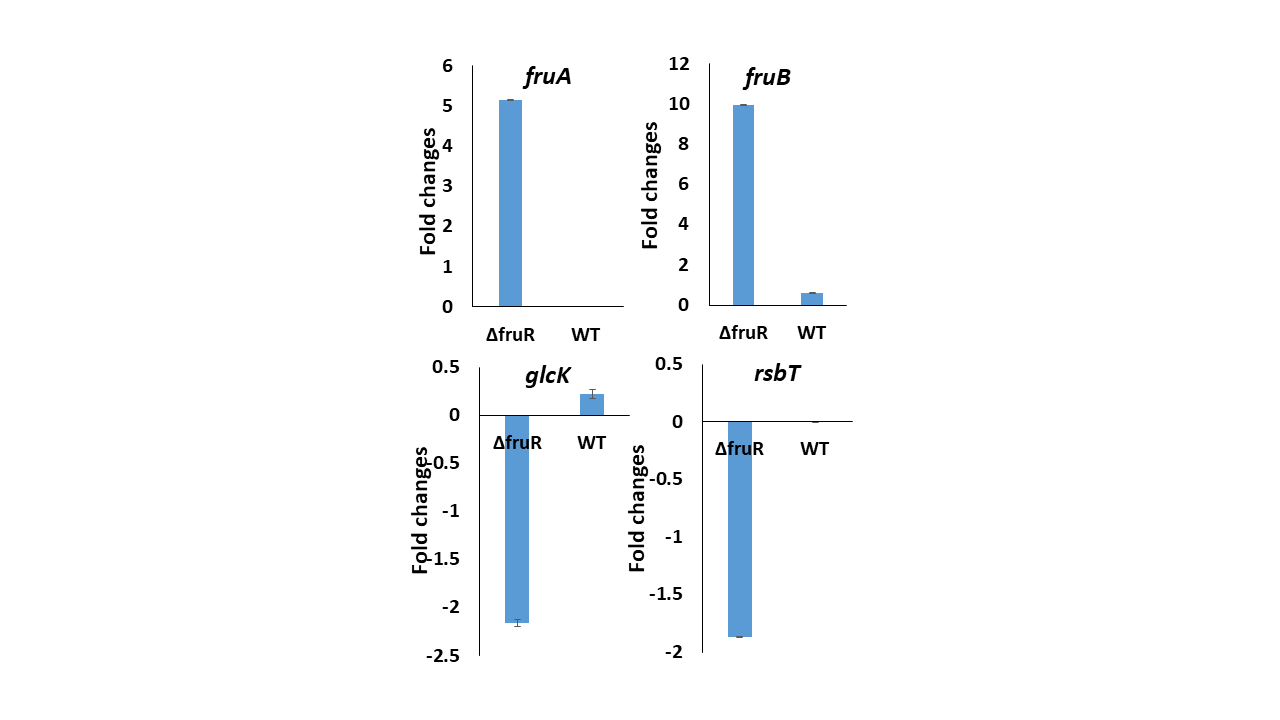

Supplement: S5 Fig — The data represent means ± standard errors from three biological replicates. (TIF) [file pone.0274005.s005.tif]

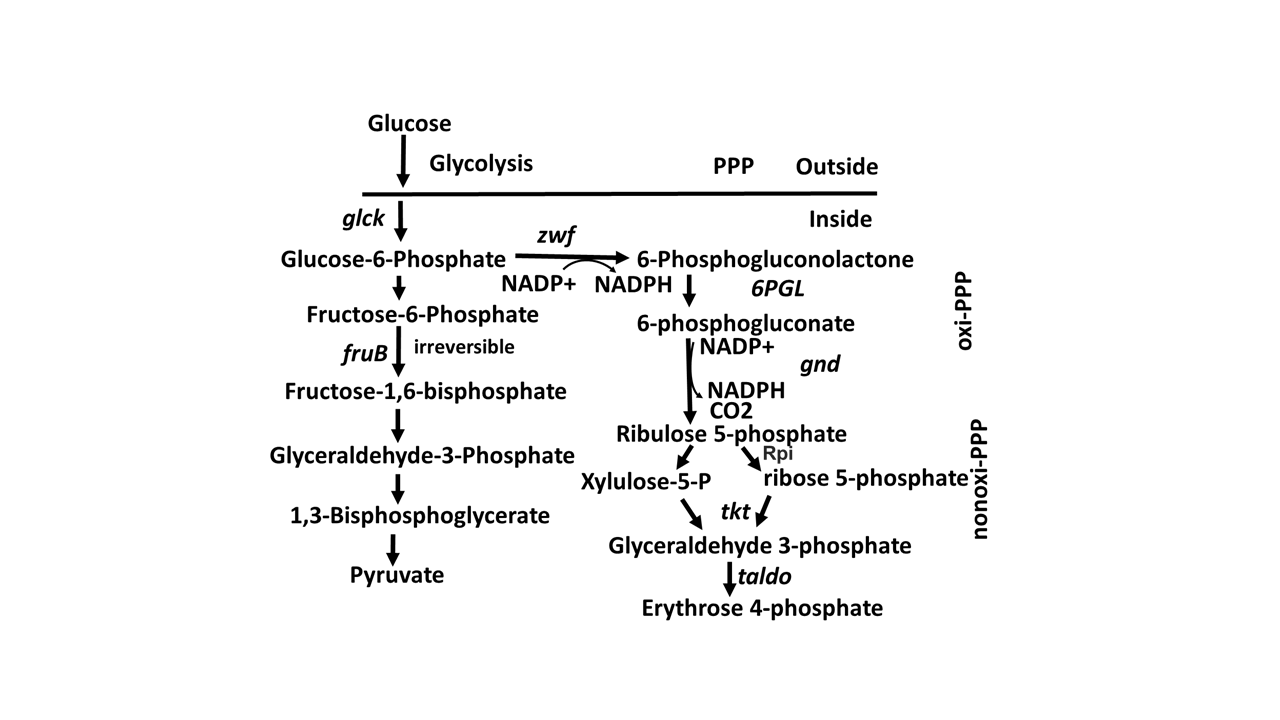

Supplement: S6 Fig — (TIF) [file pone.0274005.s006.tif]
